# GLP-1 Receptor Agonists Are Associated with Reduced Ascending Aorta Dilatation in Patients with Type 2 Diabetes: A Prospective Study

**DOI:** 10.3390/ijms26209977

**Published:** 2025-10-14

**Authors:** Celestino Sardu, Ludovica Vittoria Marfella, Carlo Fumagalli, Luca Rinaldi, Ferdinando Carlo Sasso, Domenico Cozzolino, Francesco Nappo, Ausilia Sellitto, Ciro Romano, Caterina Carusone, Pasquale Russo, Lorenza Marfella, Nicola Maria Tarantino, Gerardo Carpinella, Fulvio Furbatto, Sandro Gentile, Giuseppina Guarino, Ersilia Satta, Alessandro Bellis, Luca Marinelli, Isabella Donisi, Nunzia D’Onofrio, Ciro Mauro, Salvatore Cappabianca, Maria Luisa Balestrieri, Raffaele Marfella

**Affiliations:** 1 Department of Advanced Medical and Surgical Sciences, University of Campania “Luigi Vanvitelli”, 80126 Naples, Italy; ludovicavittoria.marfella@studentiunicampania.it (L.V.M.); carlo.fumagalli@unicampania.it (C.F.); luca.rinaldi@unimol.it (L.R.); ferdinandocarlo.sasso@unicampania.it (F.C.S.); domenico.cozzolino@unicampania.it (D.C.); francesco.nappo@policliniconapoli.it (F.N.); ausilia.sellitto@policliniconapoli.it (A.S.); ciro.romano@unicampania.it (C.R.); caterina.carusone@policliniconapoli.it (C.C.); celestinosardu@libero.it (P.R.); lorenza.marfella@studentiunicampania.it (L.M.); raffaele.marfella@unicampania.it (R.M.); 2 Pollution and Cardiovascular Diseases Research Centre, University of Campania “Luigi Vanvitelli”, 81100 Caserta, Italy; nunzia.donofrio@unicampania.it (N.D.); marialuisa.balestrieri@unicampania.it (M.L.B.); 3 Department of Precision Medicine, University of Campania “Luigi Vanvitelli”, 80138 Naples, Italy; nicolamaria.tarantino@unicampania.it (N.M.T.); luca.marinelli@unicampania.it (L.M.); isabella.donisi@unicampania.it (I.D.); salvatore.cappabianca@unicampania.it (S.C.); 4 Department of Cardiology, AORN Antonio Cardarelli, 80131 Naples, Italy; gerardocarpinella@gmail.com (G.C.); fulvio.furbatto@aocardarelli.it (F.F.); alessandro.bellis@aocardarelli.it (A.B.); ciro.mauro@aocardarelli.it (C.M.); 5 Department of Clinical Research, Nefrocenter Research Network, Via XXV Luglio, 160, 84013 Cava de Tirreni, Italy; sarducelestino@libero.it (S.G.); dr.giuseppinaguarino@libero.it (G.G.); ersilia.satta.fuvg@napoli.omceo.it (E.S.)

**Keywords:** type 2 diabetes mellitus, ascending aortic dilatation, GLP-1 receptor agonists, computed tomography angiography, vascular biomarkers, inflammation

## Abstract

The aim was to assess the impact of glucagon-like peptide-1 receptor agonists (GLP-1 RA) treatment on the progression of ascending aorta dilatation in patients with type 2 diabetes mellitus (T2DM). A total of 127 T2DM patients with subclinical ascending aortic dilatation (35–45 mm) were prospectively enrolled. Fifty-seven initiated GLP-1 RA therapy (liraglutide, semaglutide, or dulaglutide), while 70 continued on standard care. Ascending aortic diameter was measured by computed tomography angiography (CTA) at baseline and 24 months, alongside circulating markers of vascular remodeling: matrix metalloproteinase-9 (MMP-9), tissue inhibitor of metalloproteinases-1 (TIMP-1), C-reactive protein (CRP), and osteoprotegerin (OPG). Progression of aortic dilatation was significantly lower in the GLP-1 RA group compared with controls (+0.36 ± 0.20 mm vs. +1.05 ± 0.28 mm; *p* < 0.001). Therapy correlated with decreased MMP-9 and CRP (*p* < 0.01) and increased TIMP-1 and OPG (*p* < 0.05). The use of GLP-1 RA was an independent predictor of low progression, even in multivariate models after adjusting for demographic, metabolic, and biomarker data. GLP-1 RA therapy was associated with reduced progression of ascending aortic dilatation in T2DM, supporting a potential vasoprotective role beyond glucose lowering.

## 1. Introduction

An increased incidence of cardiovascular complications, including both microvascular and macrovascular, is well-documented in type 2 diabetes mellitus (T2DM). Among these, ascending aortic dilatation represents an important but underrecognized complication. The ascending aorta is particularly vulnerable because of its proximity to the left ventricle and exposure to pulsatile hemodynamic forces. Structural remodeling is driven by chronic inflammation, oxidative stress, and extracellular matrix (ECM) degradation, often mediated by elevated matrix metalloproteinases (MMPs), especially MMP-2 and MMP-9 [1,2]. The formation of an ascending aorta does not appear to be overt; however, the progressive dilation affects the increased rigidity of the arterial system, systolic hypertension, and left ventricular preload, resulting in the aggravation of cardiovascular risk in diabetes [3]. Recent developments in diabetes treatments have shown that glucagon-like peptide-1 receptor agonists (GLP-1 RAs) have potential cardiovascular protective benefits in addition to their glycemic benefits. Several large cardiovascular outcome trials (CVOTs) [4,5,6], including LEADER, REWIND, and SUSTAIN-6, have demonstrated reductions in major adverse cardiovascular events (MACE) in patients exposed to GLP-1RAs. Mechanistically, GLP-1 RAs have been demonstrated to prevent inflammation, mitigate oxidative stress, and regulate vasculature remodeling enzymes, such as MMP-9 and tissue inhibitors of metalloproteinases (TIMPs), in preclinical models [7,8,9,10]. In addition, GLP-1 RAs have been shown to have a possible impact on the formation of aneurysms and on aortic wall degradation, especially in models of abdominal aortic aneurysm (AAA) [11,12]. Nevertheless, the effect of GLP-1 RAs on the development of ascending aortic dilatation in individuals with T2DM has not been fully investigated. Advanced imaging modalities, such as CTA, offer accurate aortic dimensional measurements, enabling the evaluation of the aortic structural impact of pharmacological interventions. Additionally, circulating biomarkers of aortic remodeling, including matrix metalloproteinase-9 (MMP-9), tissue inhibitor of metalloproteinase-1 (TIMP-1), C-reactive protein (CRP), and osteoprotegerin (OPG), provide further insight into the biological mechanisms and progression of the disease [12,13,14,15]. Based on the known anti-inflammatory and anti-remodeling effects of GLP-1 RAs, we hypothesize that treatment with these agents may resist the progression of ascending aortic dilatation in T2DM patients. In the current study, we aimed to investigate the impact of GLP-1 RA therapy on the structural development of ascending aortic dilatation over a 24-month follow-up period, as assessed through serial CTA imaging, and to understand the relationship with changes in circulating vascular remodeling markers. This study may provide new insights into the vasoprotective role of GLP-1 RAs and expand their therapeutic implications in the prevention of diabetes-associated macrovascular complications.

## 2. Results

### 2.1. Baseline Characteristics

The baseline demographic and clinical characteristics of the study participants (N = 127) are presented in Table 1. Of those, 57 newly GLP-1 RA-treated (guidelines-based regimen: suboptimal glycemic control and/or overweight/obese) patients were enrolled. The other 70 patients, who did not meet the above criteria, were treated with conventional antidiabetic therapy and were defined as the control group. There were more males in the general population (63.0%), with a mean age of 63.8 ± 6.9 years and a mean history of diabetes of 9.9 ± 3.0 years. Not surprisingly, the GLP-1 RA group had a higher mean BMI at baseline than the control group, consistent with the indication for therapy. The mean HbA1c was also marginally higher in the GLP-1 RA group compared to controls (*p* = 0.03). Other cardiometabolic characteristics were well-balanced between groups. The baseline ascending aortic diameter on contrast-enhanced CTA did not differ at a significant level between groups (Table 1).

### 2.2. Follow-Up Clinical Monitoring and Cardiometabolic Evolution

All subjects completed the intended 24 months of follow-up through in-person visits at 6-monthly intervals. These assessments included the following: physical examination, a review of therapy, ECG, carotid Doppler ultrasound (TSA), and transthoracic echocardiography (TTE). Both groups demonstrated high treatment adherence, with over 90% of participants continuing with the treatment at the end of the study. As presented in Table 2, significant changes in major cardiometabolic outcomes were observed in the GLP-1 RA group across the various time periods. In patients on GLP-1 RA, a mean BMI decrease from 32.5 ± 3.2 to 29.6 ± 3.0 kg/m^2^ was noted (*p* < 0.001), i.e., an approximately 8% weight loss over 24 months. In comparison, the BMI in the control group showed no significant change (*p* = 0.22). The mean HbA1c decreased in both groups. The MAP decreased in the GLP-1 RA group (*p* = 0.002) but not significantly more than in the control group (*p* < 0.001).

**Figure 1:**
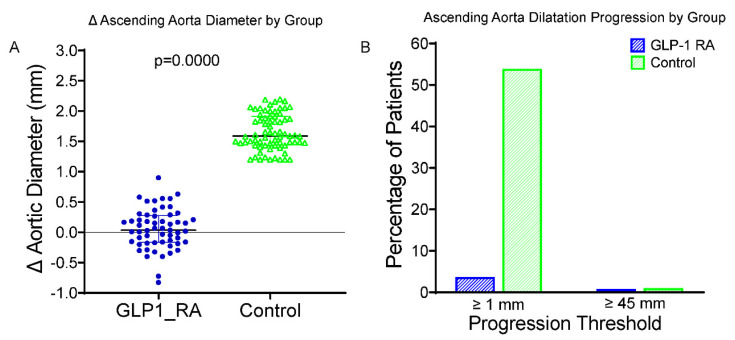
Changes in and progression of ascending aorta diameter in GLP-1 RA vs. control groups. (**A**) Boxplot of change (Δ) in ascending aortic diameter over 24 months in patients treated with GLP-1 RAs and controls. The GLP-1 RA group vs. controls showed significantly less progression (*p* < 0.0001). (**B**) Bar graph showing the proportion of patients with progression (Δ ≥ 1.0 mm, **left**) or reaching ≥45 mm diameter at follow-up (**right**). A symbolic bar (0.5%) was used where no events occurred. GLP-1 RA = glucagon-like peptide-1 receptor agonist; Δ = change from baseline; mm = millimeters; *p* = *p*-value from independent-samples *t* test.

A significant reduction in LDL cholesterol concentration was observed in both groups. At 24 months, the increase in ascending aortic dilatation in patients receiving GLP-1 RAs was significantly less than that in the control group (Figure 1A). The average changes in the diameter of the ascending aorta were +0.38 ± 0.23 mm in the GLP-1 RA group and +1.13 ± 0.31 mm in the control group (*p* < 0.001). Two patients (3.5%) of the GLP-1 RA group, and forty (57.1%) of the control subjects exhibited the enlargement of ≥1.0 mm in ascending aorta diameter (Figure 1B). Importantly, only one patient (1.8%) in the GLP-1 RA group and one patient (1.4%) in the control group surpassed the clinical cut-off of 45 mm. This difference was not statistically significant, reflecting the small number of events. These results highlight that the protective effect of GLP-1 RA treatment on aortic wall remodeling for individuals with T2DM is independent of its beneficial influence on glycemic and blood pressure regulation. Excellent intraobserver and interobserver reproducibility were found for the aortic diameter measurements (intraclass correlation coefficient [ICC] > 0.90); for the latter, the minimal detectable change was established at 0.2 mm, thus attesting to the reliability of the anatomical data.

Conversely, in the GLP-1 RA group, the HbA1c decreased significantly from 8.4 ± 0.6% to 7.1 ± 0.5% and mean arterial pressure from 98.4 ± 7.2 mmHg to 91.5 ± 6.7 mmHg over 24 months (both *p* < 0.01), whereas the reductions in the control group were less pronounced. Importantly, in multivariable analyses adjusting for these changes, GLP-1 RA therapy remained an independent predictor of reduced aortic dilatation, indicating that the observed vascular benefits were not solely mediated by glycemic or blood pressure control.

### 2.3. Circulating Biomarker Changes

**Figure 2:**
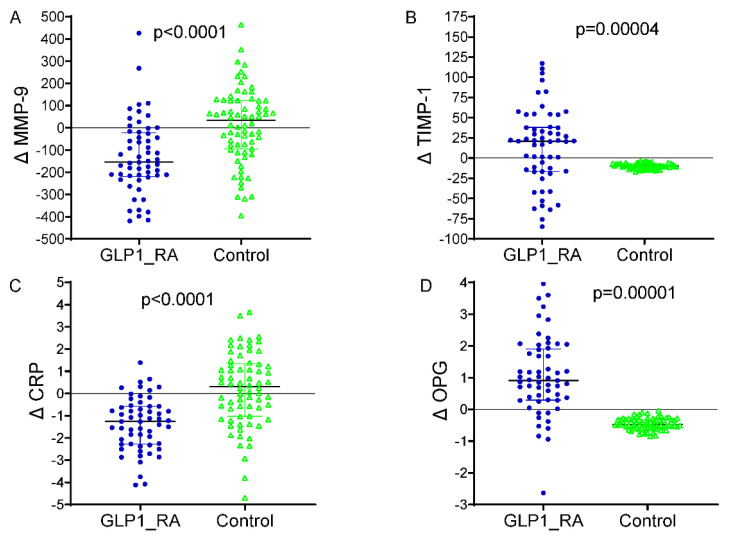
Changes in circulating biomarkers from baseline to 24 months by treatment group. (**A**) Δ MMP-9: significant reduction in the GLP-1 RA group vs. controls (*p* < 0.0001). (**B**) Δ TIMP-1: increased in GLP-1 RA and decreased in controls (*p* = 0.00004). (**C**) Δ CRP: marked reduction with GLP-1 RA therapy (*p* < 0.0001). (**D**) Δ OPG: increased in GLP-1 RA and decreased in controls (*p* = 0.00001). GLP-1 RA = glucagon-like peptide-1 receptor agonist; MMP-9 = matrix metalloproteinase-9; TIMP-1 = tissue inhibitor of metalloproteinases-1; CRP = C-reactive protein; OPG = osteoprotegerin; Δ = change from baseline. *p* = *p*-value from independent-samples *t* test. In blue color the representation of delta values for MMP-9, TMP-1, CRP and OPG in the patients treated with GLP-1 RA. In green colour the delta values for MMP-9, TMP-1, CRP and OPG in the patients without GLP-1RA therapy (controls).

**Figure 3:**
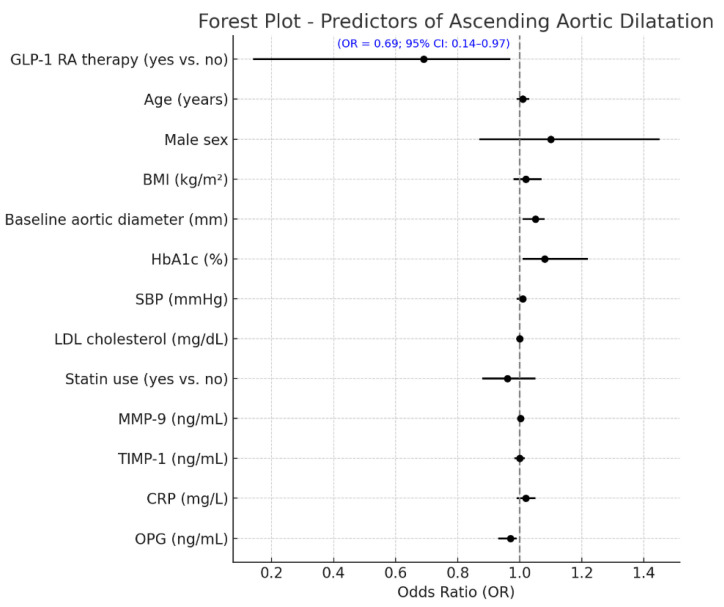
Independent predictors of aortic arch dilatation progression at 24 months. Forest plot showing independent predictors of aortic arch progression (≥1.0 mm increase over 24 months) from multivariate logistic regression. Variables included baseline characteristics, biomarker changes, and GLP-1 RA use. GLP-1 RA therapy was linked to a significantly lower risk of progression (OR = 0.57; 95% CI: 0.14–0.97; *p* = 0.010). Other significant predictors included Δ BMI and Δ MMP-9 (both with trends toward significance). Odds ratios are shown with 95% confidence intervals; the red dashed line (OR = 1.0) marks no effect. GLP-1 RA = glucagon-like peptide-1 receptor agonist; OR = odds ratio; CI = confidence interval; BMI = body mass index; SBP = systolic blood pressure; Δ = change from baseline; MMP-9 = matrix metalloproteinase-9; TIMP-1 = tissue inhibitor of metalloproteinases-1; CRP = C-reactive protein; OPG = osteoprotegerin.

The differences between baseline and 24 months in circulating vascular biomarkers are presented in Figure 2. A marked decrease in MMP-9 levels was observed in patients receiving GLP-1 receptor agonists, which dropped from 556.2 ± 123.4 ng/mL at baseline to 421.1 ± 113.2 ng/mL at the end of 24 months (*p* < 0.001). The TIMP-1 increased from 161.4 ± 33.7 ng/mL to 175.5 ± 34.7 ng/mL (*p* = 0.003), with the CRP decreasing from 4.1 ± 1.2 mg/L to 2.8 ± 0.7 mg/L (*p* < 0.001). An increase in the OPG was demonstrated as well, from 3.9 ± 0.9 ng/mL to 4.9 ± 1.0 ng/mL (*p* = 0.002). The control group did not significantly increase MMP-9 levels over the infusion, from 558.8 ± 124.4 ng/mL to 570.2 ± 141.8 ng/mL (*p* = 0.04), and the TIMP-1 was virtually unchanged (156.7 ± 34.4 to 160.4 ± 18.2 ng/mL, *p* = 0.31). The mean (+s.d.) CRP increased, but the difference was not significant, from 3.9 ± 1.2 to 4.2 ± 1.3, mg/L (*p* = 0.11), and the OPG did not change significantly and was 3.8 ± 0.6, ng/mL in both periods (*p* = 0.21). Between-group comparisons of the 24-month Δ values revealed significant differences for all biomarkers (ΔMMP-9, ΔTIMP-1, ΔCRP, and ΔOPG), corroborating the premise that GLP-1 RA therapy exerts a beneficial modulation on vascular inflammation and ECM remodeling. Furthermore, Table 3 shows that the multivariate linear regression model analyzing predictors of change in ascending aortic diameter over 24 months revealed that GLP-1 RA treatment was an independent factor associated with a markedly reduced progression in aortic dilatation (*p* < 0.001) when controlling for clinical and biochemical covariates. The baseline aortic diameter, along with the treatment effect, was an additional significant predictor (*p* = 0.004), such that the larger the initial aortic diameter, the more likely dilatation would subsequently occur. Larger aortic expansion was also seen in those with more poorly controlled DM (β = +0.081; 95% CI +0.018 to +0.144; *p* = 0.015) at baseline, highlighting the importance of controlling DM in regard to vascular remodeling. Of the circulating biomarkers, increased levels of MMP-9 (*p* = 0.001) and lower levels of TIMP-1 (*p* = 0.008) were significantly associated with a larger aortic diameter, suggestive of a matrix degradation profile. In addition, a lower OPG (*p* = 0.043) was independently associated with higher progression. The final model explained approximately 47% of the variation in ascending aortic dilatation (adjusted R^2^ = 0.47), indicating a strong predictive ability of both clinical and biochemical factors in determining aortic structural abnormalities in patients with T2D. A multivariable logistic regression model was built to identify independent predictors of aortic arch diameter progression, defined as a 24-month diameter increase of ≥1.0 mm. The response variable was dichotomous (1 = progression, 0 = stability or decrease). The ultimate model also adjusted for clinical covariates (age, sex, systolic blood pressure, baseline HbA1c, and statin use), changes in circulating biomarkers (ΔMMP-9, ΔTIMP-1, ΔCRP, and ΔOPG), and metabolic features (decreases in BMI and HbA1c during follow-up). In this model, aortic dilatation progression was independently associated with a significantly reduced odds of treatment with GLP-1 receptor agonists (OR 0.69, 95% CI 0.14–0.97, *p* = 0.010) (Figure 3). An independent protective effect was also noted for MMP-9; OR: 1.002 per unit change (95% CI: 1.000–1.005; *p* = 0.099). TIMP-1 was also not independently associated with aortic outcomes (OR: 0.999; 95% CI: 0.982; 1.017; *p* = 0.927). Reductions in BMI and HbA1c during follow-up displayed a tendency towards a higher risk of fracture: with each 1 kg/m^2^ decline in BMI, the OR determined was 1.106 (95% CI: 0.996–1.228; *p* = 0.060), and for a 0.5% decline in HbA1c it was 1.317 (95% CI: 0.613–2.830; *p* = 0.480), neither of which was statistically significant. Baseline HbA1c and statin use were not significant in the multivariable model. Model calibration and discrimination were confirmed, and no multicollinearity was observed among the covariates.

### 2.4. Sensitivity Analyses

To assess the robustness of our findings, several sensitivity analyses were performed. When considering the ascending aortic diameter as a continuous variable, the adjusted mean difference between groups remained statistically significant (β = −0.62 mm; 95% CI −0.73 to −0.50; *p* < 0.001). The effect persisted when alternative cut-off thresholds for progression were applied. Using a more sensitive threshold (≥0.5 mm increase), progression was observed in 28.1% of the GLP-1 RA group and 71.4% of controls (OR = 0.19; 95% CI 0.09–0.38; *p* < 0.001). Excluding biomarker changes from the multivariate models yielded consistent results (OR = 0.21; 95% CI, 0.10–0.44; *p* < 0.001), thereby reducing the potential for overadjustment bias. Moreover, inverse probability weighting based on a propensity score incorporating demographic and metabolic covariates confirmed the association between GLP-1 RA therapy and reduced aortic dilatation (β = −0.58 mm; 95% CI, −0.70 to −0.45; *p* < 0.001). These findings support the robustness of the observed association across different analytic strategies.

### 2.5. Cardiac Safety and Efficacy

There was no statistically significant MACE during the moderating follow-up period in the GLP-1 RA group. In addition, only four events were recorded in the control group: two non-fatal myocardial infarctions, one ischemic stroke, and one hospitalization for heart failure (5.7 vs. 0%; *p* = 0.045). There were no deaths in either group. GLP-1 RA was well tolerated, with mild gastrointestinal side effects (nausea, early satiety) present in 14.0% of patients; discontinuation of therapy was not necessary in any of them.

## 3. Discussion

In this prospective study, we provide the first evidence that GLP-1 RA treatment is independently associated with a decreased progression of aortic arch dilatation in T2DM. In patients under GLP-1 RA therapy, neither structural enlargement of the aortic arch (measured by contrast-enhanced CTA) nor changes in circulating biomarkers related to vascular remodeling and inflammation increased over 24 months. Our most novel finding is that GLP-1 RA treatment, known to be effective for glycemic control and reducing CVD risk, may also produce direct structural benefits on the thoracic aorta. This is in addition to the evidence derived from cardiovascular outcome trials (CVOTs), such as LEADER, SUSTAIN-6, and REWIND, which have demonstrated lower rates of MACE in patients treated with GP-1 RA [4,5,6]. Although these trials have focused on anti-atherosclerotic and endothelial-stabilizing effects, our findings may extend to exposure of a stringent endpoint involving aortic wall integrity and structural progression. Crucially, the structural improvements detected in our study were mechanistically associated with a discriminative biomarker profile indicative of anti-inflammatory and anti-remodeling properties. In particular, GLP-1 RA treatment was associated with a reduction in the plasma levels of MMP-9 and CRP, and an increase in TIMP-1 and OPG. These molecular alterations are particularly relevant to the pathogenesis of aortic dilatation. MMP-9 is a matrix peptidase derived from the zinc component that is involved in the extracellular matrix proteolysis and is directly linked with aneurysm development and passing away in both the infrarenal and thoracic aortas [7,8,9,10,11,12,13,14,15]. In contrast, TIMP1, a natural MMP inhibitor, is known to have a function in stabilizing the vessel extracellular matrix [13]. The balance between MMP-9 and TIMP-1 is thus considered essential for VSMC remodeling during vascular injury. In our multivariate analysis, lower MMP-9 and higher levels of TIMP-1 at follow-up were independent predictors of lesser aortic progression, supporting the notion that inhibition of matrix degradation is a major pathway for reducing vascular dilatation. Likewise, CRP, a classic acute-phase protein and a circulating systemic inflammatory marker, was markedly reduced with GLP-1 RA treatment, reinforcing the anti-inflammatory benefits of this therapeutic class. The contribution of chronic low-grade inflammation to the progression of aortic dilatation is gradually being appreciated; indeed, CRP levels have been proven to be associated with aortic stiffness, medial degeneration, and elastin fragmentation [15]. Of interest, the biomarker OPG, part of the TNF receptor superfamily, also rose during GLP-1 RA treatment. While OPG functions in both the pro- and anti-vascular biology processes, it also has immune regulatory effects. In this case, higher OPG levels could also represent a compensatory vascular protective mechanism [14]. Here, the biomarker findings were in line with previous preclinical studies, which have shown that treatment with a GLP-1 RA attenuates aortic aneurysm in mice, in part through the regulation of MMP levels and by preventing NF-κB-driven inflammation [11,12,13,14,15,16,17,18]. However, until now, there has been a lack of human data in this regard. Our work presents new clinical evidence of remodeling of the thoracic aorta following the use of GLP-1 RA. It contributes to the expanding literature in favor of the vascular effects of this therapeutic category. The consequences of these observations are profound. Aortic arch dilatation is a precursor for potentially catastrophic events, which are aneurysm rupture and dissection. Even incremental changes in diameter, such as those found in the control group in the present study (+1.13 mm over 2 years), are associated with increased aortic stiffness, left ventricular afterload, and an increased risk of subsequent heart failure, as well as in diabetic subjects [16,17].

Accordingly, treatments that influence aortic remodeling may exert considerable lifelong cardiovascular benefits [18,19]. Our findings suggest that GLP-1 RAs may have a unique potential to provide such protection independently of their effects on glycemia or body weight. Of note, whilst both GLP-1 RA and control groups received beneficial effects on traditional cardiometabolic parameters (HbA1c, blood pressure, LDL cholesterol), only recipients of GLP-1 RA consistently improved on the structural/marker level. This underscores the possibility of direct vasodilatory effects of GLP-1 RAs that are independent of conventional risk factor modification. Indeed, GLP-1 RA use was a strong independent predictor of reduced aortic progression in multivariable regression models adjusting for baseline MRA (Table 3), HbA1c, and statin therapy, as well as for blood pressure and LDL cholesterol. Clinically, this could have an impact on future risk stratification and treatment of patients with subclinical aortic dilatation and type 2 diabetes mellitus (T2DM). At present, there are no drugs that are specifically recommended for the prevention of the formation of thoracic aortic enlargement in diabetic patients. Our data could support the idea of considering GLP-1 RA as drug therapy to control and modulate aortic enlargement, especially in patients with borderline aortic diameters and raised biomarkers of matrix degradation. Moreover, our study included several sensitivity analyses to address potential biases inherent to the observational design. Specifically, the association between GLP-1 RA therapy and attenuated aortic dilatation remained significant across multiple definitions of progression (≥0.5 mm and ≥1.0 mm increase), supporting the consistency of the findings. Furthermore, the effect persisted after adjusting for relevant clinical confounders and excluding changes in circulating biomarkers to minimize the risk of overadjustment bias. Importantly, a propensity score–weighted regression confirmed that GLP-1 RA use remained independently associated with reduced aortic enlargement after accounting for differences in baseline characteristics, including age, BMI, glycemic control, and lipid profile. These additional analyses reinforce the robustness and validity of the observed association, although causality cannot be inferred. Although our primary endpoint was a surrogate anatomical marker (millimetric change in ascending aortic diameter), this measurement is clinically relevant, since even small increments have been associated with increased aortic stiffness, left ventricular afterload, and future cardiovascular risk in T2DM patients [3,4,5,6,7]. Finally, our data confirm that GLP-1 RA therapy improves multiple cardiometabolic risk factors, including weight, glycemic control, blood pressure, and lipid profile, which may partially mediate the attenuation of aortic dilatation [3,4,5,6,7]. Notably, even after adjusting for these variables, GLP-1 RA therapy remained an independent predictor of reduced progression, suggesting additional direct vasoprotective effects. In this setting, the GLP-1 RA could modulate complex and multiple molecular and cellular pathways (MMP-9, TIMP-1, CRP, and OPG), thus supporting the concept of a dual benefit, where both metabolic improvements and anti-remodeling mechanisms contribute to stabilizing the aortic wall. Notably, GLP-1 RA therapy improved both glycemic control and blood pressure during follow-up, consistent with the expected cardiometabolic benefits of this drug class. However, these improvements did not fully explain the attenuation of aortic dilatation, since GLP-1 RA treatment remained independently associated with reduced progression after adjusting for changes in HbA1c and mean arterial pressure. This suggests that the protective effect on the aortic wall extends beyond conventional risk factor modification and is likely mediated by direct anti-inflammatory and anti-remodeling mechanisms, as reflected by the biomarker profile. Furthermore, although the absolute difference in ascending aortic diameter progression observed over 24 months was modest, this finding should not be underestimated. Prior studies have shown that even small incremental increases in thoracic aortic diameter are linked to greater aortic stiffness, increased left ventricular afterload, and elevated cardiovascular risk in patients with T2DM [3,4,5,6,7]. In our study, the millimetric attenuation of dilatation associated with GLP-1 RA therapy was consistently supported by parallel changes in the biomarkers of vascular remodeling and inflammation (reduced MMP-9 and CRP and increased TIMP-1 and OPG), reinforcing the biological plausibility of a true structural protective effect. Importantly, while few patients reached the conventional threshold of ≥45 mm within the relatively short follow-up, the consistent attenuation of progression suggests that GLP-1 RA therapy may exert long-term vasoprotective benefits, particularly in patients with borderline aortic diameters or high-risk biomarker profiles. Nonetheless, the clinical impact of these small structural changes requires further exploration. Furthermore, despite the robustness of our findings across multivariable models, sensitivity analyses, and propensity score weighting, residual confounding remains possible. As this was a prospective observational study, unmeasured or incompletely captured factors (e.g., lifestyle variables, concomitant therapies, genetic predispositions) may still have influenced both treatment allocation and vascular outcomes. Therefore, our results should be interpreted as associations rather than proof of causality. This limitation underscores the need for randomized controlled trials to confirm whether GLP-1 RA therapy exerts a direct protective effect on aortic remodeling and to establish whether the observed structural changes translate into improved clinical outcomes. Future randomized controlled trials are warranted to further delineate the mechanistic pathways and to determine whether these structural benefits translate into improved cardiovascular outcomes. Nonetheless, several limitations warrant consideration. First, while our sample size and follow-up duration are among the largest to date for imaging-based studies in this area, the cohort remains relatively modest and from a single center. Indeed, our study was conducted at a single center in Naples, Italy, with a relatively modest sample size (n = 127). The limited sample size may have reduced statistical power for subgroup analyses, raising the possibility of type II error and underscoring the need for confirmation in larger adequately powered studies. However, while this design allowed uniform imaging protocols and biomarker analyses, it may limit the external validity and generalizability of our findings to other populations and healthcare settings. Second, although we adjusted for multiple confounders, residual bias cannot be excluded in this observational design. Indeed, patients receiving GLP-1 RA therapy had higher BMI and HbA1c at baseline, reflecting guideline-driven treatment allocation. Moreover, although adjusted and propensity score-weighted analyses were performed, residual bias related to these differences cannot be entirely excluded. Third, our study focused exclusively on thoracic (ascending aorta) dilatation; whether similar benefits would be observed in the abdominal aorta remains unknown. Conversely, the primary endpoint relied on imaging-based surrogate measures of aortic diameter progression, without prespecified clinical outcomes (such as aneurysm rupture, dissection, or need for surgical repair), and no significant differences were observed for the hard threshold of ≥45 mm due to the relatively short follow-up and small number of events. In this setting, only a very small number of patients exceeded the clinical threshold of >45 mm during follow-up, which limits conclusions about the direct clinical impact. However, the consistent attenuation of progression and associated biomarker changes suggest potential long-term benefits that merit confirmation in larger cohorts.

Future research should aim to validate our findings in larger multi-center cohorts and assess whether GLP-1 RAs can prevent clinical aortic events, such as aneurysm rupture or the need for surgical repair. Serial imaging of both the thoracic and abdominal aorta in response to GLP-1 RA therapy may provide a more comprehensive understanding of vascular remodeling dynamics in diabetes. In addition, mechanistic studies exploring the impact of GLP-1 RAs on endothelial function, vascular smooth muscle cell phenotype, and collagen/elastin architecture may yield important insights to add to current knowledge [19,20]. Ultimately, these results highlight the importance of integrating imaging and biomarker strategies in assessing vascular health in diabetes. The simultaneous assessment of structural and molecular changes offers a powerful platform for monitoring disease progression and therapeutic response. Taken together, our findings suggest a potential protective association between GLP-1 RA therapy and attenuation of ascending aortic dilatation in T2DM; however, these results should be regarded as hypothesis-generating and require confirmation in larger multicenter randomized trials before any implications for clinical guidelines can be drawn. Larger multicenter studies with more diverse cohorts will be necessary to validate these observations and establish their broader applicability.

## 4. Methods and Materials

This was a prospective observational cohort study conducted in the Internal Medicine outpatient units of the University of Campania “Luigi Vanvitelli” (Naples, Italy), spanning the period from patient enrolment in January 2019 to the completion of follow-up in March 2024. The study was approved by the local ethics committee, and all included patients provided informed consent. The principles of the Declaration of Helsinki were followed in the conduct of the study.

### 4.1. Study Population

A total of 127 consecutive adult patients with type 2 diabetes mellitus (T2DM), from 40 to 75 years old, were recruited. All patients had subclinical ascending aorta dilatation, which was defined as an ascending aorta of 35 to 45 mm in diameter when contrast-enhanced computed tomography angiography (CTA) was performed for cardiovascular risk stratification. This dimension is in accordance with early-stage dilatation in non-syndromic high cardiovascular risk adults, which is also supported by the echocardiographic and imaging criteria about mild dilatation [14]. Patients who met the following criteria were eligible to participate: diagnosis of T2DM for ≥5 years, an HbA1c of 7.0–10.0%, and on a stable antihypertensive and lipid-lowering regimen for ≥3 months before study entry. Key exclusion criteria were previous or current GLP-1 receptor agonist use, known aortic aneurysm or dissection, history of CVD events in the last 6 months, severe renal dysfunction (eGFR < 45 mL/min/1.73 m^2^), chronic inflammatory or autoimmune disease, active malignancy, or contraindications to iodinated contrast media. Treatment groups were allocated according to clinical standards at the constituting time point. Fifty-seven patients (who qualified according to the guidelines for initiation of GLP-1 receptor agonist due to insufficient glycemic control and/or high cardiovascular risk) were treated with GLP-1 RA (liraglutide, semaglutide, or dulaglutide) therapy in combination with the baseline treatment. Seventy patients not meeting the GLP-1 RA guideline criteria were maintained on their current antidiabetic regimen (metformin, DPP-4 inhibitors, and/or basal insulin) and served as the control arm. In our study, all patients underwent ECG evaluation at baseline and during the scheduled follow-up visits as part of the cardiovascular monitoring protocol. ECG recordings were primarily used to exclude major arrhythmic or ischemic events.

### 4.2. Follow-Up and Clinical Evaluation

All patients have been followed up for 24 months, and regular 6-month clinical assessments have been conducted. During each follow-up visit, patients received a thorough cardiovascular evaluation, which included a physical examination performed directly by cardiologists and a 12-lead ECG, carotid duplex ultrasonography (CDU), and transthoracic echocardiography (TTE), with special interest in measuring the diameter of the ascending aorta. Metabolic indices (HbA1c, fasting glucose, lipid profile, renal function) and blood pressure were also followed. For all study groups, antidiabetic treatments were titrated according to clinical judgment and actual guidelines, and GLP-1 RA therapy was maintained without dose reduction, except in cases of adverse events. Drug intake compliance was assessed through pill counts and patient diaries. The present follow-up strategy is consistent with the 2022 ACC/AHA guidelines [14], which advise regular imaging follow-up, also for patients with dilatation of the ascending aorta, to monitor disease progression and inform treatment decisions.

### 4.3. Imaging Protocol

All patients underwent contrast-enhanced CTA of the thoracic aorta at baseline and after the 24-month follow-up. The scans were acquired with a 64-slice multidetector CT scan (LightSpeed VCT; GE Healthcare, Milwaukee, WI, USA) using a standardized protocol developed for the assessment of the ascending aorta. Iodinated contrast agent (Iohexol 350 mgI/mL) was administered intravascularly (dose, 1.2 mL/kg; flow rate, 4.0 mL/s) through an antecubital vein, and then 30 mL saline was injected into the arm according to bolus tracking. Bolus tracking was used to determine the optimal scan delay, utilizing a region of interest on the ascending aorta. Image acquisition was initiated with an attenuation threshold of 100 HU. The acquisition parameters were as follows: tube voltage 120 kVp, tube current 300–500 mAs (automatic modulation), collimation 0.625 mm, pitch 0.9, rotation time 0.5 s. Reconstructions were performed with a slice thickness of 1 mm and an interval of 0.5 mm collimation with a medium-sharp kernel. Ascending aorta dimensions were measured in the true axial plane at the level of the right pulmonary artery perpendicular to the long axis of the vessel. This is in accordance with the previously described methods for proper aortic diameter measurements [15]. All images were transferred to a workstation (Advantage Workstation 4.7, GE Healthcare, Chicago, IL, USA) for multiplanar and curved-planar reformats. Data were measured by two experienced radiologists who were unaware of the treatment group and clinical condition. Each scan was measured 3 times by each radiologist. The average final value derived from the six values (n = 3 per reader) was the final value for each patient and the given period. Intra- and interobserver agreement were determined using the ICC, where values above 0.90 were considered to represent excellent agreement. The D_mdc (in diameter) was 0.2 mm, calculated by means of repeated measurements and variance analysis. For outcome modeling, we dichotomized ascending aortic progression according to the CTA measurements. Progression was defined as the occurrence of an ascending aortic diameter dilatation of ≥1.0 mm of the ascending aorta at 24 months compared to the baseline (coded as 1). Those skulls that showed a ≤1.0 mm increase in diameter (stabilized or decreased in diameter) were considered as having no progression (thus, coded as 0). This cut-off threshold was established above the mean measurement uncertainty; so, that a progression call represented an actual anatomical change rather than simply a statistical variation. This binary outcome was used as the dependent variable in the OR regression model to determine the clinical and biochemical correlates of progressive AA dilation over time.

### 4.4. Biomarker Analysis

All participants provided venous blood samples in the morning after overnight fasting (≥10 h) at baseline and during the 24-month follow-up. Blood was taken into EDTA tubes and within 30 min of collection was centrifuged for 15 min at 1500× *g* at 4 °C. Plasma was also aliquoted and stored at −80 °C until batch analysis. The levels of the following plasma biomarkers were measured using commercially available enzyme-linked immunosorbent assay (ELISA) kits (R&D Systems, Minneapolis, MN, USA):

Matrix Metalloproteinase-9 (MMP-9) = Human Quantikine ELISA (sensitivity: 0.156 ng/mL; intra-assay CV < 6.2%; inter-assay CV < 8.7%)

Tissue Inhibitor of Metalloproteinases-1 (TIMP-1) = Human Quantikine ELISA Kit (sensitivity: 0.08 ng/mL; intra-assay CV > 5.4%; inter-assay CV > 7.1%)

C-Reactive Protein (CRP) = high sensitive CRP ELISA Kit (sensitivity: 0.1 mg/L; intra-assay CV < 4.5%; inter-assay CV < 6.3%)

Osteoprotegerin (OPG) = Human OPG DuoSet ELISA Kit (sensitivity 0.05 ng/mL, intra-assay CV < 6.0%, inter-assay CV < 9.0%).

Each measurement was performed in duplicate. Clinical and imaging data were concealed to laboratory operators. Calibration curves with the recombinants’ standards were provided by the manufacturer for each analyte, and concentrations were determined by 4-parameter logistic curve fit. All specimens with out-of-bound values were required to be re-run after excessive dilution. The baseline and follow-up samples of each participant were analyzed in the same batch and run to reduce the inter-assay variation. Hemolyzed and freeze–thawed degraded samples were not included. QC samples were incorporated in every batch to monitor the inter-batch variation. In the last dataset, we retained only patients with good paired biomarkers at both sampling times.

### 4.5. Statistical Analysis

The baseline characteristics and outcomes of the study were described and summarized using descriptive statistics. Continuous variables were presented as means ± standard deviation (SD) or medians and interquartile ranges (IQR) to the method of distribution based on the Shapiro–Wilk test. Categorical data are described as numbers with percentage frequency. Between-group comparisons were conducted through the independent-samples *t* test or Mann–Whitney U test for continuous variables and χ^2^ test or Fisher’s exact test for dichotomous variables. Paired *t* tests or Wilcoxon signed-rank tests were employed to analyze the changes from baseline to 2 years in the ascending aorta diameter and biomarker concentrations within groups, and independent-samples tests were used to analyze changes between groups. The reproducibility of aortic measurements was evaluated by the intraclass correlation coefficient (ICC), and the minimal detectable change (MDC) was obtained according to the formula of repeated measurements. In order to investigate the relationship between the clinical variables and ascending aorta dilatation, a multivariate linear regression model was first used with the change in diameter of the ascending aorta as the dependent variable. The independent variables were age, BMI, sex, baseline ascending aorta diameter, HbA1c, systolic blood pressure, LDL cholesterol, statins, and levels at follow-up of MMP-9, TIMP-1, CRP, and OPG. Furthermore, a multivariable logistic regression model was developed for the determination of the independent predictors of progression of ascending aorta dilatation, as a binary endpoint: • Progression = 1 for participants who had increase in the ascending aorta by 1.0 mm or more at 24 months. • No progression = 0 = stable or decreased diameter (<1.0 mm) in patients with follow-up. The potential covariates for the logistic regression model included age, sex, baseline diameter of AA, HbA1c, systolic blood pressure, statin use, Δ of biomarkers (MMP-9, TIMP-1, CRP, OPG), and changes in BMI and HbA1c from baseline to follow-up. The findings are presented as odd ratios (ORs) with 95% confidence intervals (CIs) and related *p* value. Model calibration and discrimination were by evaluated the standard diagnostics, and multicollinearity was eliminated. To evaluate the robustness of the primary findings, multiple sensitivity analyses were conducted. First, the effect of GLP-1 RA therapy on ascending aortic diameter progression was re-examined using alternative thresholds for defining progression (≥0.5 mm and ≥1.0 mm increase). Second, a sensitivity analysis was performed by excluding patients in the upper tertile of the baseline aortic diameter (>43 mm) and by stratifying the regression models according to the baseline HbA1c (above vs. below median), BMI tertiles, and sex. Interaction terms between the treatment group and each covariate were included to assess the effect modification. Collinearity was checked using variance inflation factors (VIF), and the model fit was evaluated using adjusted R^2^ and residual analysis. Third, multivariate logistic regression models were repeated excluding changes in circulating biomarkers to mitigate potential overadjustment bias. All tests were two-sided, and a value of *p* < 0.05 was considered significant. Statistical analyses were conducted in IBM SPSS Statistics (version 28.0; IBM Corp., Armonk, NY, USA) and R (version 4.2.1; R Foundation for Statistical Computing, Vienna, Austria).

## Figures and Tables

**Table 1:** Baseline characteristics of patients receiving GLP-1 receptor agonists (GLP-1 RA group) versus those on standard antidiabetic therapy (control group). Data are reported as mean ± SD or n (%). Differences in BMI and HbA1c reflect guideline-based eligibility for GLP-1 RA initiation. No other significant differences were observed between groups. GLP-1 RA, glucagon-like peptide-1 receptor agonist; BMI, body mass index; HbA1c, glycated hemoglobin; MAP, mean arterial pressure; LDL, low-density lipoprotein; eGFR, estimated glomerular filtration rate; ACEi, angiotensin-converting enzyme inhibitor; ARB, angiotensin receptor blocker; SGLT2i, sodium–glucose co-transporter-2 inhibitor. Baseline demographic, clinical, and therapeutic characteristics of the study population.

Variable	GLP-1 RA Group (n = 57)	Control Group (n = 70)	*p* Value
Age, years	63.4 ± 6.8	64.1 ± 7.0	0.48
Male sex, n (%)	36 (63.2%)	44 (62.9%)	0.97
BMI, kg/m^2^	32.5 ± 3.2	28.1 ± 2.9	<0.001
Duration of diabetes, years	10.1 ± 2.9	9.8 ± 3.1	0.57
HbA1c, %	8.4 ± 0.6	8.1 ± 0.7	0.03
Systolic blood pressure, mmHg	134 ± 11	136 ± 12	0.38
Mean arterial pressure, mmHg	98.4 ± 7.2	99.1 ± 6.9	0.61
LDL cholesterol, mg/dL	98.4 ± 13.7	96.7 ± 14.1	0.47
eGFR, mL/min/1.73 m^2^	84.6 ± 12.5	86.1 ± 11.8	0.37
eGFR ≥ 90, n (%)	20 (35.1%)	27 (38.6%)	0.71
eGFR 60–89, n (%)	34 (59.6%)	39 (55.7%)	0.68
eGFR < 60, n (%)	3 (5.3%)	4 (5.7%)	0.92
Hypertension, n (%)	43 (75.4%)	52 (74.3%)	0.88
Dyslipidemia, n (%)	40 (70.2%)	51 (72.9%)	0.72
History of cardiovascular disease, n (%)	11 (19.3%)	13 (18.6%)	0.92
Statin use, n (%)	43 (76.1%)	55 (78.6%)	0.73
ACEi/ARB use, n (%)	38 (66.7%)	49 (70.0%)	0.69
Beta-blocker use, n (%)	19 (33.3%)	25 (35.7%)	0.78
Antiplatelet therapy, n (%)	21 (36.8%)	26 (37.1%)	0.97
Metformin use, n (%)	51 (89.5%)	65 (92.9%)	0.52
SGLT2i use, n (%)	17 (29.8%)	14 (20.0%)	0.21
Insulin use, n (%)	13 (22.8%)	15 (21.4%)	0.85
Aortic arch diameter, mm	38.3 ± 2.4	38.1 ± 2.2	0.48

**Table 2:** Cardiometabolic and vascular biomarker changes over 24 months in patients with type 2 diabetes treated with GLP-1 receptor agonists vs. standard therapy. Data are presented as mean ± SD or number (%). *p* values refer to comparisons within each group (baseline vs. follow-up) and between groups at 24 months. BMI, body mass index; HbA1c, glycated hemoglobin; BP, blood pressure; LDL, low-density lipoprotein cholesterol; eGFR, estimated glomerular filtration rate, MMP-9, matrix metalloproteinase-9; TIMP-1, tissue inhibitor of metalloproteinases-1; CRP, C-reactive protein; OPG, osteoprotegerin.

Parameter	GLP-1 RA Group Baseline	GLP-1 RA Group 24 mo	*p* (Within Group)	Control Group Baseline	Control Group24 mo	*p* (Within Group)	*p* (Between Groups at 24 mo)
BMI, kg/m^2^	32.5 ± 3.2	29.6 ± 3.0	<0.001	28.1 ± 2.9	27.9 ± 3.0	0.22	<0.001
HbA1c, %	8.4 ± 0.6	7.1 ± 0.5	<0.001	8.1 ± 0.7	7.2 ± 0.5	<0.001	0.047
MAP, mmHg	98.4 ± 7.2	91.5 ± 6.7	0.002	99.1 ± 6.9	90.8 ± 6.8	<0.001	0.78
LDL cholesterol, mg/dL	98.4 ± 13.7	86.1 ± 11.9	<0.001	96.7 ± 14.1	90.6 ± 12.3	0.012	0.041
Aortic ascending diameter, mm	38.3 ± 2.4	38.7 ± 2.5	<0.001	38.1 ± 2.2	39.2 ± 2.3	<0.001	<0.001
MMP-9, ng/mL	556.2 ± 123.4	421.1 ± 113.2	<0.001	558.8 ± 124.4	570.2 ± 141.8	0.04	<0.001
TIMP-1, ng/mL	161.4 ± 33.7	175.5 ± 34.7	0.003	156.7 ± 34.4	160.4 ± 18.2	0.31	0.005
CRP, mg/L	4.1 ± 1.2	2.8 ± 0.7	<0.001	3.9 ± 1.2	4.2 ± 1.3	0.11	<0.001
OPG, ng/mL	3.9 ± 0.9	4.9 ± 0.8	0.002	3.7 ± 0.6	3.8 ± 0.6	0.21	0.004

**Table 3:** Multivariate linear regression—predictors of ascending aorta progression. Abbreviations: GLP-1 RA = glucagon-like peptide-1 receptor agonist; BMI = body mass index; BP = blood pressure; LDL = low-density lipoprotein; HbA1c = glycated hemoglobin; MMP-9 = matrix metalloproteinase-9; TIMP-1 = tissue inhibitor of metalloproteinases-1; CRP = C-reactive protein; OPG = osteoprotegerin; CI = confidence interval.

Variable	β Coefficient	95% CI	*p* Value
GLP-1 RA therapy (yes vs. no)	−0.72	(−0.94 to −0.51)	<0.001
Age (years)	+0.010	(−0.004 to +0.024)	0.16
Male sex	+0.092	(−0.074 to +0.258)	0.27
BMI (kg/m^2^)	+0.014	(−0.007 to +0.035)	0.19
Baseline aortic diameter (mm)	+0.046	(+0.017 to +0.075)	0.004
HbA1c (%)	+0.081	(+0.018 to +0.144)	0.015
Systolic BP (mmHg)	+0.005	(−0.002 to +0.013)	0.13
LDL cholesterol (mg/dL)	+0.001	(−0.004 to +0.006)	0.72
Statin use (yes vs. no)	−0.039	(−0.185 to +0.108)	0.60
MMP-9, ng/mL	+0.004	(+0.001 to +0.006)	0.001
TIMP-1, ng/mL	−0.006	(−0.010 to −0.002)	0.008
CRP, mg/L	+0.020	(−0.006 to +0.046)	0.13
OPG, ng/mL	−0.028	(−0.056 to −0.001)	0.043

## Data Availability

The data that support the findings of this study are available from the corresponding author upon reasonable request.
